# Amelioration of BPSD-Like Phenotype and Cognitive Decline in SAMP8 Mice Model Accompanied by Molecular Changes after Treatment with I_2_-Imidazoline Receptor Ligand MCR5

**DOI:** 10.3390/pharmaceutics12050475

**Published:** 2020-05-23

**Authors:** Foteini Vasilopoulou, Andrea Bagan, Sergio Rodriguez-Arevalo, Carmen Escolano, Christian Griñán-Ferré, Mercè Pallàs

**Affiliations:** 1 Pharmacology Section, Department of Pharmacology, Toxicology and Medicinal Chemistry, Faculty of Pharmacy and Food Sciences, and Institut de Neurociències, University of Barcelona, Av. Joan XXIII, 27-31, E-08028 Barcelona, Spain; ftn.vasilopoulou@gmail.com; 2 Laboratory of Medicinal Chemistry (Associated Unit to CSIC), Department of Pharmacology, Toxicology and Medicinal Chemistry, Faculty of Pharmacy and Food Sciences, and Institute of Biomedicine (IBUB), University of Barcelona, Av. Joan XXIII, 27-31, E-08028 Barcelona, Spain; abaganpo7@alumnes.ub.edu (A.B.); rodriguez.arevalo@ub.edu (S.R.-A.); cescolano@ub.edu (C.E.)

**Keywords:** I_2_-imidazoline receptors, Behavioural and psychological symptoms of dementia (BPSD), brain-derived neurotrophic factor (BDNF), neuroinflammation, age

## Abstract

Behavioural and psychological symptoms of dementia (BPSD), including fear-anxiety- and depressive-like behaviour, are present in Alzheimer’s disease (AD), together with memory decline. I_2_-imidazoline receptors (I_2_-IRs) have been associated with neuropsychiatric and neurodegenerative disorders, further, I_2_-IR ligands have demonstrated a neuroprotective role in the central nervous system (CNS). In this study, we assessed the effect of the I_2_-IR ligand MCR5 on both cognitive and non-cognitive symptoms in the Senescence accelerated mice prone 8 (SAMP8) mouse model. Oral administration of I_2_-IR ligand MCR5 (5 mg/kg/day for four weeks) in 10-month SAMP8 mice ameliorated both BPSD-like phenotype and cognitive decline by attenuating depressive-like behaviour, reducing fear-anxiety-like behaviour and improving cognitive performance using different tasks. Interaction of I_2_-IR ligand MCR5 with serotoninergic system did not account for behavioural or cognitive improvement, although changes in molecular pathways underlying depression and anxiety phenotype were observed. MCR5 increased levels of p-AKT, phosphorylated glycogen synthase kinase 3 β (GSK3β) at Ser9 and phosphorylated mammalian target of rapamycin complex 1 (mTORC1) levels in SAMP8 treated mice compared to SAMP8 control. Moreover, MCR5 treatment altered N-methyl-d-aspartate receptor (NMDA) 2B phosphorylation, and decreased the protein levels of phosphorylated cyclin-dependent kinase 5 (p-CDK5) and dopamine- and cyclic adenosine monophosphate (cAMP)-regulated phosphoprotein of Mr 32 kDa phosphorylated at Thr75 (p-DARPP32), with a parallel increase in protein kinase A (PKA) and p-cAMP response element-binding (pCREB) levels. Consistent with these changes MCR5 attenuated neuroinflammation by decreasing expression of pro-inflammatory markers such as *Tumor necrosis factor-alpha (Tnf-α), Interleukin 1β (Il-1β), Interleukin 6 (Il-6),* and promoted synaptic plasticity by increasing levels of postsynaptic density protein 95 (PSD95) as well as ameliorating tropomyosin-related kinase B (TrkB) and nerve growth factor receptor (NGFR) signalling. Collectively, these results increase the potential of highly selective I_2_-IR ligands as therapeutic agents in age-related BPSD and cognitive alterations.

## 1. Introduction

Nowadays, the global population of the elderly is increasing in parallel to the diagnosis of neurodegenerative diseases (ND) and psychiatric disorders. Thus, ageing is the main factor associated with ND, such as Alzheimer’s disease (AD), which is the leading cause of dementia [1]. So far, the AD research has mainly focused on cognitive impairment and typical AD hallmarks, although no effective treatments are able to reduce or halt the progression of the disease [2].

On the other hand, non-cognitive symptoms are becoming increasingly important due to their prevalence, generating several dysfunctions and representing one of the most troublesome domains of treating dementia [3]. These non-cognitive symptoms that patients suffer, commonly referred to as “Behavioural and psychological symptoms of dementia” (BPSD), mainly include aberrant motor behaviour, hallucinations, aggressive and anxiety behaviour and depression, among others [4]. In AD, the patients present BPSD since the early stages [5], which, as the disease is progressing, tend to increase their frequency. BPSD are the cause of higher morbidity and poor quality of life for patients and caregivers [6]. Approximately between 40% and 60% of AD patients experience anxiety and depressive symptoms [7]. Therefore, these symptoms are associated with cognitive impairment, increasing the risk of persistence for mild cognitive impairment (MCI) and dementia, being both the most common psychiatric syndromes [8,9].

The biogenic monoamine 5-hydroxytryptamine (5-HT), or serotonin, has been tied to cognitive decline and multiple other BPSD domains [10,11], while it is well-known that loss in serotonin transporters and changes in synaptic proteins can fire mechanisms linked to depression by alteration of specific cerebral circuits [12]. Several studies support that the mechanisms underlying the complex manifestations of anxiety and depressive illness involve dysregulation of brain-derived neurotrophic factor (BDNF) [13,14] and N-methyl-d-aspartate receptor (NMDAR) [15], promoting disturbances in cellular signalling and neuronal plasticity. Among cellular pathways affected in the pathophysiology of depression, we can name the mammalian target of rapamycin (mTOR), mitogen-activated protein kinases (MAPK) or glycogen synthase kinase 3 (GSK-3), as discussed.

I_2_-Imidazoline receptors (I_2_-IRs) have been progressively attracting scientific interest as a promising biological target, which modulation could result in potential therapeutic effects. Several preclinical studies describe I_2_-IRs involvement in neurological diseases such as AD, Parkinson’s disease (PD) and multiple psychiatric disorders [16,17,18]. I_2_-IRs have been associated with anxiety and depressive behaviours [16,19]. In preclinical models of mood disorders, several I_2_-IR ligands exerted beneficial effects on non-cognitive symptoms [20,21]. Interestingly, agmatine, the endogenous I_2_-IR ligand [22], has a modulatory effect on anxiety and depression [23,24]. Specifically, those studies that had utilized allegedly α2-selective imidazoline radioligands, i.e., [^3^H]-clonidine, could be reinterpreted in terms of the increased number of those receptors in depression. Although the molecular identity of the I_2_ binding site remains unknown, an I_2_ binding site has been reported to be encoded by monoamine oxidase genes (both MAO-A and MAO-B), suggesting a novel explanation for the antidepressant efficacy of idazoxan, a prototypic I_2_-IR ligand. We have previously described, MCR5 diethyl [(1-(3-chloro-4-fluorobenzyl)-5,5-dimethyl-4-phenyl-4,5-dihydro-1*H*-imidazol-4-yl]phosphonate] as an I_2_-IR highly selective compound over α2-adrenoreceptors and high affinity for the I_2_-IR [25], characterized by neuroprotective abilities in models of cognitive decline and AD [26,27].

The senescence-accelerated mouse prone 8 (SAMP8) is an accelerated ageing model, established through phenotypic selection from a common genetic pool of AKR/J-strain of mice [28,29,30]. Besides the age-related cognitive decline that mainly characterizes the SAMP8 model, this strain also displays anxiety- and depressive-like behaviour in comparison to their senescence-acceleration resistant counterparts, SAMR1 mice [28]. In particular, the SAMP8 mouse presents alterations in the serotoninergic system and BDNF expression, calcium signalling pathways and increased neuroinflammation that lead to the development of BPSD and in particular depressive-like behaviour [31]. Collectively, therefore it is believed that this rodent model is appropriate for studying depressive-like behaviour in an aged population with cognitive impairment.

In the present study, we assessed the cognitive and non-cognitive effects, especially anxiety- and antidepressant-like effects as the main BPSD behaviours of the I_2_-IR ligand MCR5 diethyl [(1-(3-chloro-4-fluorobenzyl)-5,5-dimethyl-4-phenyl-4,5-dihydro-1*H*-imidazol-4-yl)phosphonate] in vivo, providing as well that the evidence referred to induced molecular changes that could explain such effects, using the SAMP8 mice model. As aforementioned, MCR5 tested in previous works of our group, presented neuroprotective and analgesic effects, showed promising results in models of brain damage [26] and prevented cognitive decline in SAMP8 aged female mice, including molecular changes associated with age and neurodegenerative processes [27]. Thus, we studied some molecular pathways related to neurodegeneration and neuropsychiatric disorders that are characteristic of age-related behavioural and cognitive abnormalities of SAMP8.

## 2. Methods

### 2.1. Animals

**Figure 1:**
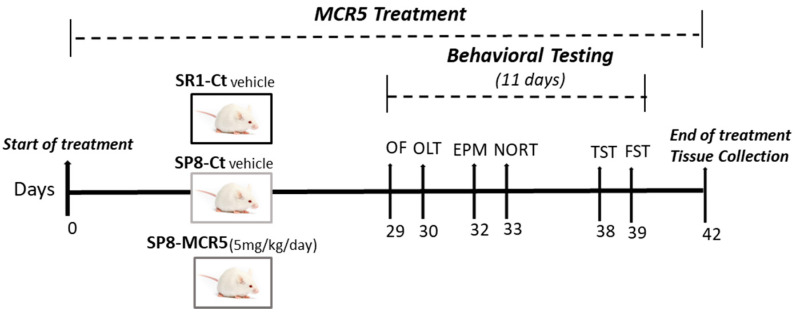
Scheme of experimental design.

SAMR1 (*n* = 11) and SAMP8 (*n* = 25) male mice (10-month-old) were used to perform behavioural and molecular analyses. The animals were divided randomly into three groups: SAMR1 Control (SR1-Ct) (*n* = 11), SAMP8 Control (SP8-Ct) (*n* = 11) and SAMP8 treated with I_2_-IR ligand MCR5 (5 mg/Kg) (SP8-MCR5) (*n* = 14). Animals had free access to food and water and were kept under standard temperature conditions (22 ± 2 °C) and a 12-h light/dark cycle (300 lux/0 lux). Control groups received water plus vehicle (1.8% 2-hydroxypropyl-β-cyclodextrin). MCR5 (5 mg/Kg/day) was dissolved in vehicle and administered through drinking water for 4 weeks. Water consumption was controlled each week, and I_2_-IR ligand concentrations in water were adjusted accordingly to reach the optimal dose (see Figure 1).

All experimental procedures involving animals followed the standard ethical guidelines of European Communities Council Directive 86/609/EEC and by the Institutional Animal Care and Use Committee of the University of Barcelona (670/14/8102, approved at 11/14/2014) and by Generalitat de Catalunya (10291, approved 1/28/2018).

### 2.2. Evaluation of Anxiety- and Depressive-Like Behaviour as Well as Cognitive Performance

#### 2.2.1. Tail Suspension Test (TST)

Briefly, to evaluate the potential anti-depressant effect of MCR5 in mice. Animals were suspended by their tail leads to an immobile posture using adhesive tape and hung approximately 30 cm above the table. The fragments, 17 cm each, of tape, were cut and an imprint 2 cm, on each fragment, was placed from one end. The task lasts for 6 min, and the duration of immobility was evaluated manually. Passively hanging was considered as immobility. The total time of mobility was subtracted from the 6 min of task time and was declared as the immobility time [32,33].

#### 2.2.2. Forced Swimming Test (FST)

The cylindrical tank (10 cm internal diameter, 50 cm high) filled with water (10 cm height) at 22–25 °C required for mice forced to swim for 6 min. The mice behaviour to avoid the aversive situation was recorded during this time. The session was videotaped, and the time that each mouse remained mobile was entirely analysed. The total time of mobility was subtracted from the 6 min of task time and was called the immobility time. The mice were considered as immobile when they keep floating, doing only those movements necessary to maintain their heads out of the water [34].

#### 2.2.3. Elevated Plus Maze (EPM)

The anxiety-related behaviour was assessed by elevated plus maze (EPM) [35]. The apparatus consisted of two open arms (30 × 5 × 15 cm), and two enclosed arms (30 × 5 × 15 cm) positioned 40 cm above the ground. The junction of four arms formed a central square platform (5 × 5 cm). Each mouse was located on the central platform facing and was allowed to move freely for 5 min. The behaviour parameters evaluated were the number of entries in the open arms and the percentage of time spent in the open and closed arms, among others, scored with SMART^®^ vers.3.0 software. In addition, the anxiety index was calculated as follows: Anxiety Index = 1 − [([Open arm time/Test duration] + [Open arms entries/Total number of entries])/2] [36]. The tests were recorded using a camera attached to the roof and located above the apparatus.

#### 2.2.4. Open Field Test (OFT)

In brief, the OFT was performed using a wall-enclosed area as previously described [37]. The ground was divided into two defined as the centre and peripheral areas. Behaviour was evaluated with SMART^®^ ver.3.0 software, and each test was recorded for later evaluation using a camera located above the apparatus. Mice were located at the centre and allowed to explore the white polywood box (50 × 50 × 25 cm) for 5 min. Then, the animals were returned to their home cages, and the OFT apparatus was cleaned with 70% ethanol (EtOH). The parameters measured included centre time duration, rearings, defecations, and the locomotor activity, calculated as the sum of global distance moved in the arena for 5 min.

#### 2.2.5. Novel Object Recognition Test (NORT)

In brief, mice were located in a 90°, two-arm, 25 × 20 × 5 cm black maze. The walls could be removed for easy cleaning. Before the memory phase, a 3-day-habituation was performed in which the mice were located individually in the apparatus for 10 min. On day 4, the familiarization phase took place, in which the animals were placed in the maze in the presence of two identical, novel objects (A + A) or (B + B) located at the end of each corner arm. During this 10-min acquisition trial, the mice were allowed to explore the two identical objects. After 2 h and 24 h from that trial, the animals were submitted to a 10-min retention trial in which one of the two objects was replaced by a novel object. The behaviour of the mice was recorded during the 2 h and 24 h retention trials using a camera attached to the roof and located above the apparatus. The time spent exploring the new object (TN) and the time spent exploring the old one (TO) were evaluated manually and the discrimination index (DI) was calculated as (TN-TO)/(TN+TO) [38]. To avoid olfactory cues, 70% EtOH was used to clean the arms and objects after test.

#### 2.2.6. Object Location Test (OLT)

The test was performed in a cage (50 × 50 × 25 cm), in which three walls were white except one that was black, and lasted 3 days. On day 1, mice were familiarized to the arena for 10 min. On day 2, two identical objects (A + A) were located in front of the black wall, and the mice were freely allowed to explore both objects for 10 min (Trial 1—training phase). On day 3, after a retention period of 24 h mice were returned to the testing arena for another 10 min (Trial 2—testing phase) with one object moved to a different position (opposite direction toward the black wall) and were allowed to explore. The trials were recorded, and the object exploration time was measured manually. The time sniffing the object in the old position (PO) and the time exploring the object in the new position (TN) were evaluated. The DI defined as (PN-TO)/(PN+PO) was determined as an indicator of the cognitive performance [39]. For the elimination of olfactory cues, 70% EtOH was used to clean the testing arena after each trial.

### 2.3. Determination of Transporters, Receptors and Alterations in Molecular Pathways

#### 2.3.1. In Vitro Pharmacology: Binding Assays

The purpose of this study was to test MCR5 in Binding assays by using Eurofins radioligand assays. Briefly, binding for receptors 5-HT1A, 5-HT1B, 5-HT2A, 5-HT2B, ion channel 5-HT3 and 5-HT transporter (SERT) was studied using radioactively labelled ligands specific for each target in human recombinant cell lines (For details see Table S6). MCR5 was tested at 10 µM. MCR5 interaction was calculated as a % inhibition of the binding of a radioactively labelled ligand specific for each target. Percentages were calculated as follow: percent inhibition of control specific binding = 100 − [(measured specific binding/control specific binding) × 100]. Results showing an inhibition or stimulation higher than 50% are considered to represent significant effects of the test compounds.

#### 2.3.2. Tissue Preparation for Biochemical Analysis

Mice were euthanised by cervical dislocation 3 days after the behavioural and cognitive tests were finished. Brains were immediately dissected out from the skull. The hippocampus of each animal was separated, snap frozen in dry ice and kept at −80 °C.

For Western Blot (WB) and immunodetection, hippocampus samples were thawed and mixed in lysis buffer containing phosphatase and protease inhibitors (Cocktail II, Sigma-Aldrich). Once mixed, samples were maintained on ice for 30 min. Samples were centrifugated at 10,000× *g* for 30 min at 4 °C, and the supernatants were collected and saved at −80 °C. Total protein amounts were obtained, and the Bradford method was used to determine protein concentration.

For ELISA evaluation, samples were processed following the instructions provided by the kit manufacturer (Biosensis, Thebarton, Australia). In brief, hippocampus samples were thawed and mixed through sonication at 4 °C in 200 volumes of RIPA buffer (50 mM Tris-HCl, 150 mM NaCl, 1% (*v/v*) NP-40 and 0.5% (*w/v*) sodium deoxycholate, pH = 7.5–8) containing a protease and phosphatase inhibitor cocktail. Once mixed, samples were maintained on ice for 30 min. Sample sonication and cooling with ice were performed. Samples were centrifuged at 14,000× *g* for 30 min at 4 °C, and the supernatants were obtained and maintained at −80 °C. Protein amount was quantified through the Bradford method.

#### 2.3.3. Protein Levels Determination by Western Blot

For WB, aliquots of 15 µg of hippocampal protein were used. Protein samples were isolated by Sodium dodecyl sulphate-Polyacrylamide gel electrophoresis SDS–PAGE (8–15%) and transferred onto PVDF membranes (Millipore, Burlington, MA, USA). The membranes were blocked in 5% non-fat milk or 5% bovine serum albumin (BSA) in Tris-buffered saline (TBS) solution with 0.1% Tween 20 (TBS-T) during 1 h at room temperature. Next, the membranes were incubated overnight at 4 °C containing the primary antibodies listed in Table S1. The antibodies were dissolved in TBS-T with 5% BSA or 5% non-fat milk.

Membranes were cleaned and incubated with the secondary antibodies for 1 h at room temperature. Immunoreactive proteins were detected with a chemiluminescence-based detection kit, following the manufacturer’s protocol (ECL Kit, Millipore, Burlington, MA, USA). Digital images were collected using a ChemiDoc XRS+ System (BioRad, Hercules, CA, USA). Semi-quantitative analyses of the band intensities were carried out using ImageLab software (BioRad), and results were expressed in arbitrary units (AU). Values were normalized to Glyceraldehyde-3-phosphate dehydrogenase (GAPDH) or β-Actin.

#### 2.3.4. Determination of ProBDNF and MBDNF Levels in the Hippocampus

The hippocampal determination of pro-Brain-derived neurotrophic factor (proBDNF) and mature-Brain-derived neurotrophic factor (mBDNF) protein levels was made according the instructions of the ELISA kit (Biosensis, Thebarton, Australia). 

#### 2.3.5. RNA Extraction and Gene Expression Determination

RNA isolation from hippocampal samples was performed using the TRIzol^®^ reagent according to the manufacturer’s protocols (Bioline Reagent, Memphis, TN, USA). The yield, purity and quality of RNA were determined with a NanoDrop™ND-1000 (Thermo Scientific, Waltham, MA, USA) apparatus and an Agilent 2100B Bioanalyzer (Agilent Technologies, Santa Clara, CA, USA). RNAs with 260/280 ratios and RNA integrity number (RIN) higher than 1.9 and 7.5, respectively, were collected. Reverse Transcription-Polymerase Chain Reaction (RT-PCR) was performed as follows: 2 μg of messenger RNA (mRNA) was reverse-transcribed using the high capacity cDNA reverse transcription kit (Applied Biosystems, Foster City, CA, USA). Real-time quantitative PCR (qPCR) was employed to quantify the mRNA expression of the genes evaluated listed in Table S2.

SYBR^®^ Green real-time PCR was performed using a Step One Plus Detection System (Applied-Biosystems) employing SYBR^®^ Green PCR Master Mix (Applied-Biosystems). Each reaction mixture contained 6.75 μL of complementary DNA (cDNA) (2 μg concentration), 0.75 μL of each primer (100 nM concentration), and 6.75 μL of SYBR^®^ Green PCR Master Mix (2X).

Data were measured using the comparative cycle threshold (Ct) method (ΔΔCt), where the housekeeping gene expression *β-Actin* was used to normalize differences in sample loading and preparation. Each sample was evaluated per duplicate, and the results represent the n-fold difference of the gene expression among groups.

### 2.4. Data Acquisition and Statistical Analysis

Data are expressed as mean ± standard error of the mean (SEM). Statistical analyses were performed using One-Way analysis of variance (ANOVA) with Tukey’s post hoc tests or two-tail Student’s t-test if it was necessary through GraphPad Prism ver. 8 for Mac (GraphPad Software, San Diego, CA, USA). Statistical differences were considered significant when values of *p* were < 0.05. Statistical outliers were discriminated using Grubbs’ test and were removed from the analysis.

## 3. Results

### 3.1. I_2_-IR Ligand MCR5 Reduced Cognitive Loss in SAMP8 Male Mice

**Figure 2:**
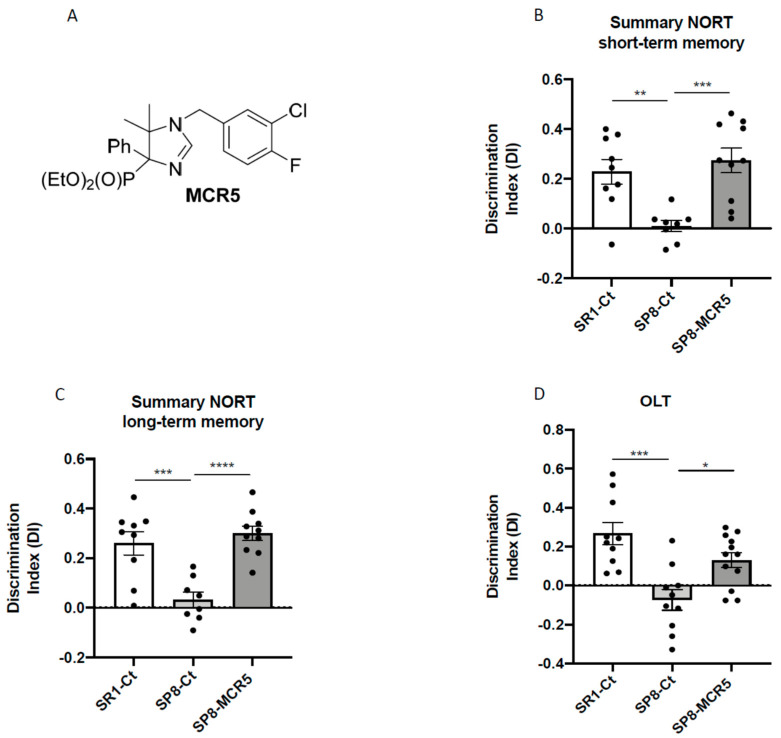
Structure of I_2_-IR ligand MCR5 (**A**). Results of Novel object recognition test (NORT), and Object location test (OLT) in male mice at 10-month-old SR1 and SP8 Ct mice groups and SP8 treated with I_2_-IR ligand MCR5 (5 mg/Kg) mice group. For NORT: Summary of Discrimination Index (DI) from short-term memory (**B**)**,** and summary of DI from long-term memory (**C**). For OLT: Summary of DI (**D**). Values represented are mean ± Standard error of the mean (SEM); *n* = 36 (SR1-Ct *n* = 11; SP8-Ct *n* = 11; SP8-MCR5 *n* = 14). * *p* < 0.05; ** *p* < 0.01; *** *p* < 0.001; **** *p* < 0.0001.

NORT and OLT revealed robust impairment in working, and spatial memory in SAMP8 control in comparison with age and sex mated SAMR1 control (Figure 2B–D). Remarkably, after treatment with MCR5, a significant increase in the discrimination index (DI) was obtained both in the NORT short-term memory test (Figure 2B) and the NORT long-term memory test in treated SAMP8 compared to the SAMP8 control group (Figure 2C). Moreover, DI for treated SAMP8 group was closer to DI delivered by SAMR1, indicating a neuroprotective effect for MCR5. Similarly, in OLT, I_2_-IR treatment significantly improved spatial memory in treated SAMP8 compared to the SAMP8 control group by increasing the DI, further demonstrating the beneficial effect of MCR5 on cognitive loss associated to age in SAMP8 mice (Figure 2D). The summary data of the NORT and OLT are presented in Supplementary Table S3.

### 3.2. I_2_-IR Ligand MCR5 Improved Emotional Parameters Associated with Fear- Anxiety- and Depressive-Like Behaviours in SAMP8 Male Mice

**Figure 3:**
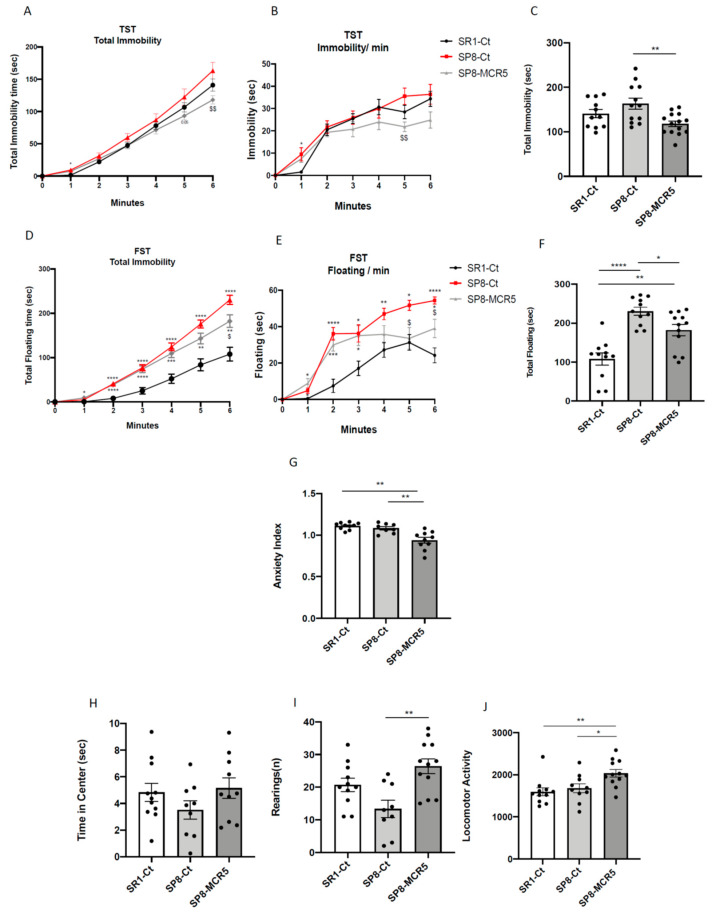
Results of Tail suspension test (TST), Forced swimming test (FST), Elevated plus maze (EPM), and Open field test (OFT) in male mice at 10-month-old SR1 and SP8 Ct mice groups and SP8 treated with I_2_-IR ligand MCR5 (5 mg/Kg) mice group. For TST: total immobility curve (**A**), immobility/min (**B**), and total immobility (**C**). For FST: total immobility curve (**D**), floating/min (**E**), and total floating (**F**). For EPM: anxiety index (**G**). For OFT: time in the centre (**H**), rearings (**I**), and locomotor activity (**J**). Values represented are mean ± Standard error of the mean (SEM); *n* = 36 (SR1-Ct *n* = 11; SP8-Ct *n* = 11; SP8-MCR5 *n* = 14). * *p* < 0.05; ** *p* < 0.01; *** *p* < 0.001; **** *p* < 0.0001.

To evaluate the anti-depressant like effect of MCR5, we assessed the TST and FST. By one hand, an increase in both immobility and floating time was obtained from the TST and FST respectively between SAMR1 and SAMP8 control groups (Figure 3A–F), corroborating the depressive-like behaviour described in SAMP8 mice. On the other hand, MCR5 treatment induced a significant decrease in time of immobility and/or floating in treated SAMP8 when compared to SAMP8 control. Therefore, in both widely used tests for the study of depression-like behaviour, we were able to observe that MCR5 treatment reversed the depressive-like signs that SAMP8 mice exhibit, driving them to a similar behaviour to SAMR1.

Behavioural and emotional changes after MCR5 treatment were evaluated by EPM and OFT. Regarding the EPM, although there were found no differences in the anxiety-like phenotype between the SAMR1 control group and SAMP8 control group, MCR5 treatment induced an anxiolytic effect on SAMP8 mice in comparison with both control groups (Figure 3G). Additional parameters measured in the EPM are presented in Supplementary Table S4. The OFT demonstrated that SAMP8 control group exhibited increased fear, presenting decreased time spent in centre as well as vertical activity or rearings (Figure 3H,I). Strikingly, MCR5 treatment induced an anxiolytic effect on SAMP8 mice in comparison with the SAMP8 control group (Figure 3H,I). Furthermore, according to the results obtained in the OFT paradigm, locomotor activity was improved in SAMP8 treated group (Figure 3J). Additional parameters and statistical scores obtained in the OFT are depicted in Supplementary Table S5. Thus, results obtained in both EPM and OFT demonstrated changes in fear-anxiety-like behaviour and locomotor activity after treatment with MCR5.

### 3.3. Interaction of I_2_-IR Ligand MCR5 with Serotonin Receptors and Transporter

Provided that the serotoninergic system is implicated in depressive behaviour, it was worth paying attention to the effect of MCR5 on serotonin receptors and transporter. Binding studies with 10 μM MCR5 to the 5-HT receptors and 5-HT transporter were performed by Eurofins (https://www.eurofinsdiscoveryservices.com/). The experiment was performed following Eurofins validation Standard Operating Procedure. The compound binding was calculated as a % inhibition of the binding of each radioactively labelled ligand specific for its target. Results indicated that MCR5 did not display a specific capacity to displace specific ligands for 5-HT_1A_, 5-HT_1B_, 5-HT_2A_, 5-HT_2B_, ion channel 5-HT_3_ and 5-HT transporter (Table S6).

**Figure 4:**
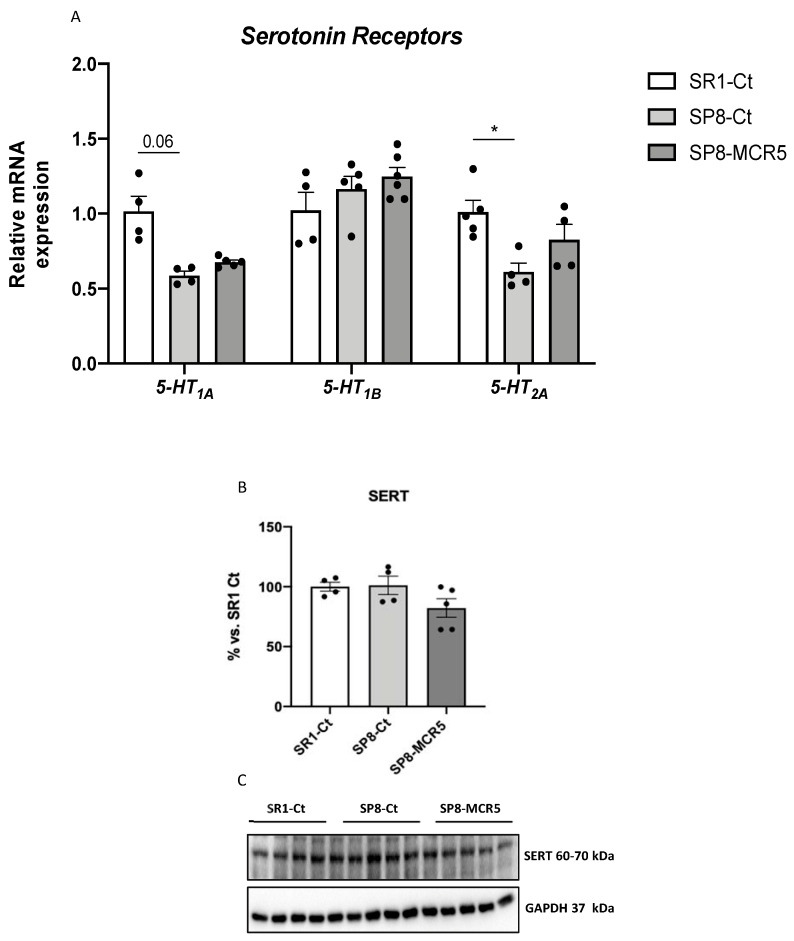
Representative gene expression for *5-HT_1A_, 5-HT_1B_,* and *5-HT_2A_* (**A**)**.** Representative Western Blot and quantification for SERT (**B**,**C**). Gene expression levels were determined by real-time PCR. Values in bar graphs are adjusted to 100% for protein levels of the control SAMR1 (SR1-Ct). Values are the mean ± Standard error of the mean (SEM); (*n* = 4–6 for each group). * *p* < 0.05.

Moreover, we studied the gene expression of *5-HT_1A_*, *5-HT_1B_* and *5-HT_2A_* receptors. As described, SAMP8 only presented a significant reduction in gene expression of *5-HT_1A_* and *5-HT_2A_* receptors compared to the SAMR1 control group (Figure 4A). Interestingly*,* treatment with MCR5 was only able to produce slight restoration of *5-HT_2A_* gene expression in SAMP8 (Figure 4A). Likewise, protein levels of SERT were unchanged between SAMR1 and SAMP8 control groups, and the MCR5 treatment did not alter the transporter’s protein levels (Figure 4B,C).

### 3.4. MCR5 Enhanced AKT/mTOR/GSK3β Pathways Promoting a Reduction of Pro-Inflammatory Cytokines in SAMP8 Male Mice

**Figure 5:**
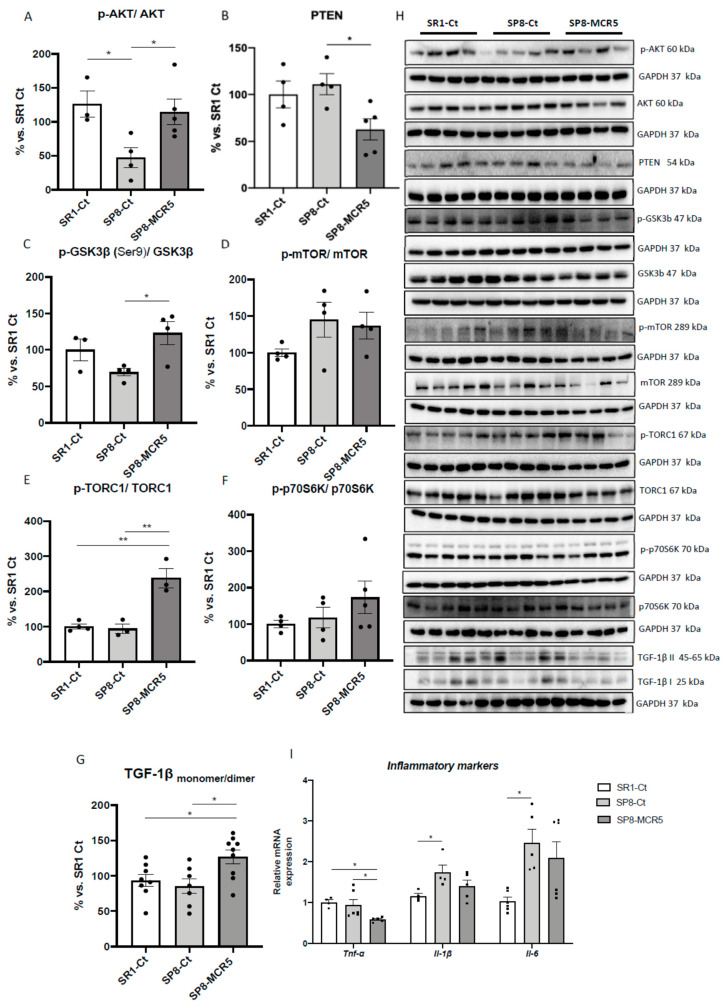
Representative Western blots and quantifications for the ratio of p-AKT/AKT, PTEN, ratio p-GSK3β(Ser9)/GSK3β, ratio p-mTOR/mTOR, ratio p-TORC1/TORC1, p-p70S6K/p70S6K, and TGF-1β monomer/dimer (**A**–**H**). Values in bar graphs are adjusted to 100% for protein levels of the control SAMR1 (SR1-Ct). Representative gene expression for *Tnf-α*, *Il-1β,* and *Il-6* (**I**). Gene expression levels were determined by real-time PCR. Values are the mean ± Standard error of the mean (SEM); (*n* = 4–6 for each group). * *p* < 0.05; ** *p* < 0.01.

Critical proteins linked to molecular pathways that are altered in the pathology of depression associated with neuronal inflammation were evaluated. WB analysis showed a significant decrease in p-AKT/AKT ratio (protein kinase B) in SAMP8 control in comparison with the SAMR1 control group. MCR5 administration to SAMP8 promoted a significant increase in p-AKT/AKT ratio compared to SAMP8 control (Figure 5A,H). We observed a slight but not significant increase in Phosphatidylinositol 3,4,5-trisphosphate 3-phosphatase (PTEN) protein levels in SAMP8 mice in comparison with SAMR1. Conversely, we found a significant diminution in PTEN levels in SAMP8 treated mice compared to the SAMP8 control group (Figure 5B,H). Accordingly, protein levels of GSK3β phosphorylated in Ser9 (p-GSK3β(Ser9)) were significantly increased after MCR5 treatment in SAMP8 mice. No significant changes between SAMR1 control and SAMP8 control were determined for this kinase (Figure 5C,H). On the other hand, p-mTOR/mTOR ratio was unaltered among the three experimental groups (Figure 5D,I). Interestingly, MCR5 increased in a significant way p-TORC1/TORC1 ratio in SAMP8 treated mice compared to both control groups (Figure 4E,H). Lastly, albeit did not reach significance, a tendency to increase p-p70S6K/p70S6K ratio after I_2_-IR ligand treatment compared to both control groups, was observed (Figure 5F,H).

Considering the results obtained on these pathways, we studied pro-inflammatory cytokines markers in the hippocampus of SAMR1 and SAMP8 mice. Significantly increased gene expression of *Il-1β* and *Il-6* in SAMP8 control in comparison with SAMR1 mice was determined, confirming the inflammatory phenotype related to anxiety- and depressive-like behaviours presented by SAMP8 (Figure 5H). A significant reduction of *Tnf-α* gene expression in MCR5 treated SAMP8 mice group compared to both control groups was found (Figure 5I). Lastly, a tendency to reduce *Il-1β* and *Il-6* gene expression in MCR5 treated SAMP8 mice group compared to the SAMP8 control group, restoring levels to those of SAMR1 control strain was found (Figure 5I). Jointly with the decrease in the proinflammatory cytokines, an increase in the anti-inflammatory cytokine ratio, TGF-1β monomer/dimer was induced by MCR5 in SAMP8 treated mice (Figure 5H,G).

### 3.5. Changes in PKA/CREB and NMDAR/CDK5/DARPP32 Signalling Cascades after Treatment with I_2_-IR Ligand MCR5 in SAMP8 Male Mice

**Figure 6:**
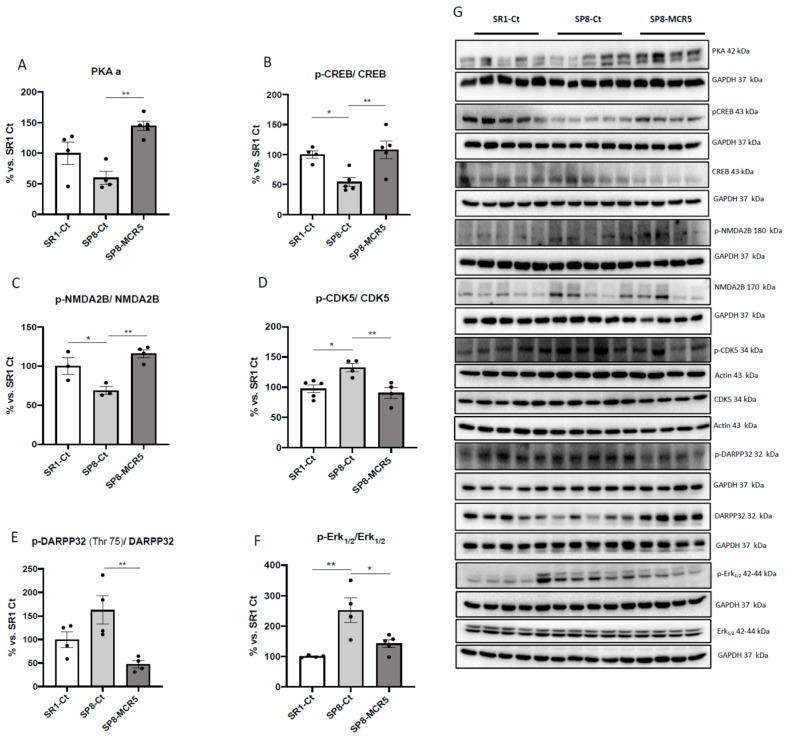
Representative Western blots and quantifications for PKA a, the ratio p-NMDA2B/NMDA2B, ratio p-DARPP32(Thr75)/DARPP32, ratio p-CDK5/CDK5, ratio p-CREB/CREB and p-Erk_1/2_/Erk**_1/2_**(**A**–**G**). Values in bar graphs are adjusted to 100% for protein levels of the control SAMR1 (SR1-Ct). Values represented are mean ± Standard error of the mean (SEM); (*n* = 4–6 for each group). * *p* < 0.05; ** *p* < 0.01.

A significant increase in PKA protein levels was found after MCR5 treatment in SAMP8 compared to untreated SAMP8 control, which expressed slightly lower levels of the mentioned kinase in comparison with SAMR1 control (Figure 6A,G). Accordingly, we found a diminished p-cAMP response element-binding (CREB)/CREB ratio in SAMP8 mice group compared to SAMR1 mice group, and MCR5 treatment restored the ratio in SAMP8 treated mice to SAMR1 levels (Figure 6B,G). A significant increase in the p-NMDA2B/NMDA2B ratio between the SAMR1 control, and SAMP8 control was observed (Figure 6C,G), and MCR5 increased p-NMDA2B/NMDA2B ratio in treated SAMP8 mice in comparison with the SAMP8 control. Moreover, we determined a significant reduction in the p-CDK5/CDK5 ratio between SAMR1 control and SAMP8 control (Figure 6D,G). Besides, a significant reduction in the p-CDK5/CDK5 ratio in SAMP8 treated mice group in comparison with the SAMP8 control mice was found. Conversely, we found reduced p-DARPP32 (Thr75)/DARPP32 ratio in the SAMR1 control group and SAMP8 treated group compared to the SAMP8 control, being significant only in the SAMP8 treated group (Figure 6E,G). Finally, we found significantly higher levels of ratio p-Erk_1/2_/Erk_1/2_ were determined in SAMP8 control mice when compared to SAMR1 control, whereas MCR5 restored p-Erk_1/2_/Erk_1/2_ to SAMR1 levels (Figure 6F,G).

### 3.6. Effects of I_2_-IR Ligand MCR5 on BDNF/TrkB/NGFR(p75) Signalling Pathway after Treatment with MCR5

**Figure 7:**
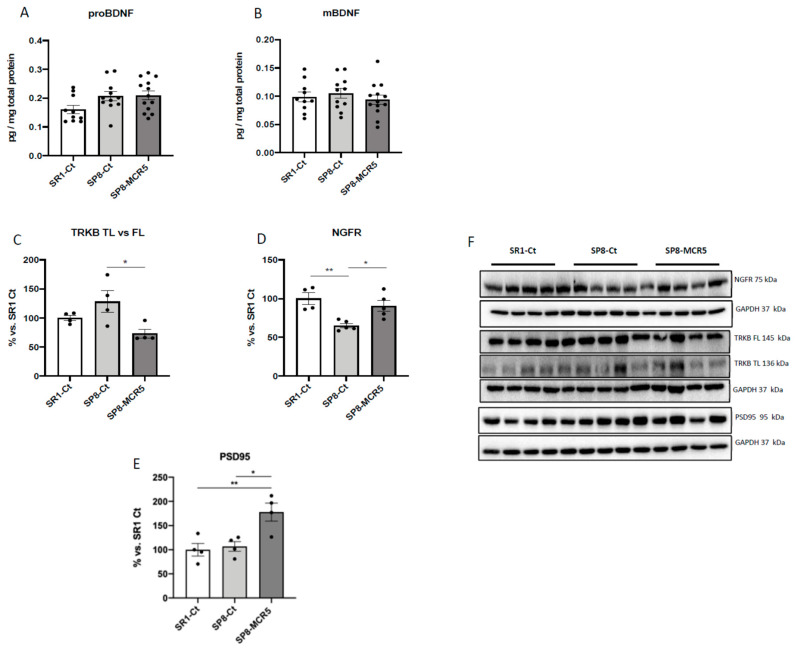
Protein levels of proBDNF (**A**), and mBDNF (**B**). Representative Western Blot and quantifications for ratio TrkB-TL vs. TrkB-FL, NGFR, and PSD95 (**C**–**F**). Values represented are mean ± Standard error of the mean (SEM); (*n* = 4–13 for each group). * *p* < 0.05; ** *p* < 0.01.

An increase in proBDNF protein levels in both SAMP8 groups in comparison with the SAMR1 control group is observed, albeit no significant differences were found (Figure 7A). Likewise, no changes in the mBDNF protein levels among experimental groups were found (Figure 7B). Pursuing BDNF molecular pathways, TrkB levels were also evaluated. Ratio TrkB-truncated (TL)/Full Length (FL) was slightly higher in the SAMP8 group compared to the SAMR1 group and was significantly reduced after MCR5 treatment (Figure 7C,F). According to these findings, a significant increase in nerve growth factor receptor (NGFR) protein levels in MCR5 treated SAMP8 in comparison with SAMP8 mice was found. Besides, a significant increase in NGFR protein levels in SAMR1 mice compared to the SAMP8 control was determined (Figure 7D,G). Lastly, we also extend the analysis of the Postsynaptic density 95 (PSD95) protein levels, a synaptic plasticity marker, which is regulated by BDNF/TrkB signalling and AKT/mTOR/GSK3β. Noteworthy, we found higher levels in SAMP8 treated with MCR5 than in control mice groups (Figure 7F,G).

## 4. Discussion and Conclusions

Besides neurodegeneration, AD has been associated with increased incidents of neuropsychiatric disorders in humans, such as anxiety and depression, among other BPSD. New research has to be performed to face this collateral complication of AD because current therapies for both AD and BPSD are not completely effective and safe. On the one hand, some AD drugs can develop BPSD signs as adverse effects, i.e., memantine. On the other hand, BPSD drugs exacerbate cognitive impairment [7].

As aforementioned, I_2_-IR are associated with the pathogenesis of several brain disorders [40,41] and neurodegenerative diseases such as AD [18]. Furthermore, I_2_-IR ligand MCR5 demonstrated neuroprotective effects under different interventions and rodent models [26,27,42]. In line with these results, MCR5 treatment improved cognitive decline presented by older SAMP8 male mice, including working and spatial memory by using NORT and OLT, respectively. The behavioural tests applied demonstrated that older SAMP8 presented, in whole, anxiety- and depressive-like behaviours, as well as fearful behaviour, with less locomotion and rears, avoiding the OF centre zone. In the present study, we showed a substantial improvement in BSPD and cognitive performance, demonstrating anti-anxiety- and anti-depressant-like effects after treatment with MCR5 in older SAMP8. Therefore, to our knowledge, this is the first study in which both changes have been demonstrated in AD mice for an I_2_-IR ligand.

Those non-cognitive and cognitive modifications promoted by MCR5 were accompanied by changes in some molecular pathways associated with ND process presented in these brain disorders. Previously it has been demonstrated that serotonin receptor densities such as 5-HT_2A_ did not suffer changes with age in SAMR1 compared to SAMP8 mice, whereas SERT increases at 9 months of age [31]. Regarding the serotoninergic system, here, we did not observe any significant change in gene expression of serotonin receptors and SERT protein levels after MCR5 treatment. Additionally, MCR5 did not bind to these membrane structures, allowing discarding an antidepressant effect mediated by inhibition of SERT or by interaction with 5-HT receptors.

It is important to note that, although the dopamine and serotonin pathways are the major targets for neuropsychiatric drugs, new mechanisms have been described, including mechanisms related to the neurodegenerative process presented in AD [43]. Indeed, it is well-demonstrated that I_2_-IR neuroprotective effects are mediated by pleiotropic mechanisms.

Accumulating evidence suggests that the pathology of main brain disorders is associated with neuronal inflammation [44,45]. It has been described that AKT/GSK3/mTOR signalling is involved in the immune cell activation, downregulating pro-inflammatory cytokines such as TNF-α, IL-1β, IL-6, and IFN-γ and upregulating the anti-inflammatory cytokines [43,46]. In addition, recent studies have demonstrated that both dopamine and serotonin exert part of their actions by modulating the activity of this pathway [47]. In this study, we reported that the I_2_-IR ligand treatment modified levels of AKT/GSK3/mTOR key proteins that might explain in part the reduction in the gene expression of the pro-inflammatory cytokine such as *Tnf-α*, *Il-1β* and *Il-6*. Conjointly with increased pro-inflammatory markers, a deficit of anti-inflammatory markers such as TGF-1β has been reported, contributing to inflammaging and cognitive impairment both in AD and other brain disorders such as depression [48,49]. I_2_-IR ligand treatment also increased levels of active TGF-1β. TGF-1β, besides Smad-mediated pathways, activates Smad-independent pathways, including the PIK3/AKT [50], further supporting the alterations observed in this pathway after I_2_ -IR treatment.

Phosphorylation of AKT protein has been shown to promote neuroprotection against cell death, increasing cell survival [51]. On the one hand, we found increased levels of p-AKT in treated SAMP8 mice in comparison to the SAMP8 control, reaching the healthy mice group, SAMR1. On the other hand, PTEN a protein that regulates AKT kinase activity was downregulated in SAMP8 treated mice. In line with our findings, fluoxetine upregulates the expression of the p-AKT [52]. Of note, SAMP8 mice showed a higher level of activated GSK3β in comparison with SAMR1 [53,54]. Moreover, some antidepressant drugs or atypical antipsychotics such as lithium regulate GSK3 by inhibiting its activity in the brain [55,56]. Changes in GSK3β activity described in SAMP8 mice reinforces the depressive-like behaviour shown. Interestingly, I_2_-IR treatment was able to decrease the GSK3β activity, giving clues for the possible pathway modulated through this receptor rending on anxiety and depression as well as improvement in cognition observed after MCR5 treatment. It can be kept in mind that inhibition of GSK3β may result in a reduction in tau hyperphosphorylation leading to the reduction in neurofibrillary tangles, then neuronal dysfunction accordingly with previous results demonstrating a neuroprotective role for MCR5 [42]. Moreover, dysregulation of mTOR signalling and concretely mTORC1, has been described under chronic restrain stress and administration of escitalopram and paroxetine prevented these changes [57]. Likewise, ketamine has been reported to activate mTOR and downstream constituents such as p70S6K resulting in increased levels of postsynaptic density proteins such as PSD95, GluR1, synapsin I [58]. In this study, we demonstrated that MCR5 treatment significantly increased ratio p-mTORC1 in SAMP8 mice in parallel with an increase in PSD95 levels, which can further explain the improvement of both cognitive and non-cognitive signs presented by SAMP8 mice.

Other signalling cascades altered after MCR5 treatment included NMDAR/CDK5/DARPP32 and PKA/CREB signalling. MCR5 inhibited NMDAR/CDK5/DARPP32 in treated SAMP8. Based on our findings, we suggest that MCR5 might exert anti-depressant-like and neuroprotective effects through this signalling pathway. DARPP32 modulates the dopamine pathway [59] and is, therefore, a key regulator for the pathogenesis of several neuropsychiatric disorders [60]. Here, we found a significant reduction of p-CDK5 and p-DARPP32 (Thr75) in SAMP8 treated with I_2_-IR ligand, implicating this pathway in the beneficial role of MCR5 in SAMP8 neuropathology. Moreover, phosphorylation of DARPP-32 in Thr75 by p-CDK5, in turn, inhibits PKA and thereby reduces the efficacy of dopamine signalling [61]. Interestingly, consistent with the reported decreased levels of p-DARPP32 in Thr75, increased PKA levels were determined in SAMP8 mice treated with I_2_-IR ligand MCR5. Indeed, we also found that MCR5 treatment induced the activation of the PKA/CREB signalling cascade, increasing protein levels of both PKA and p-CREB. Of interest, several studies demonstrated that activation of PKA/CREB pathway in the hippocampus leads to neuroprotective effects not only by upregulating BDNF protein levels, but also reducing neuroinflammation in several brain disorders such as anxiety, in an early episode of depression and AD [62]. This evidence is coincident with the diminution of pro-inflammatory cytokines such as *Tnf-α*, *Il-1β* and *Il-6* found after MCR5 administration. Given its implication in CREB phosphorylation, we also investigated the levels of p-Erk_1/2_. Surprisingly, its levels were found increased in SAMP8 control mice and significantly decreased in the SAMP8 treated mice. However, increased ERK_1/2_ activation has been reported in socially defeated animals, and Erk_1/2_ mediated increase in inflammatory markers is established [63,64,65]. In the present study, a reduction of Erk_1/2_ activation induced by MCR5 treatment is also consistent with the decreased pro-inflammatory markers observed.

Given that downregulation of BDNF function has been demonstrated in the brains of patients with neurodegenerative or neuropsychiatric disorders, evaluation of the BDNF levels and TrKB signalling in the hippocampus after MCR5 treatment was relevant [13,14]. Indeed, one cause for the reduced BDNF levels is due to BDNF/TrkB signalling dysfunction mediated by endogenous small molecules, driving to changes associated with the above-mentioned pathways [66,67]. In our hands, MCR5 treatment did not produce any change in proBDNF and mBDNF protein levels. However, MCR5 ameliorated BDNF/TrkB signalling, promoting TrkB-FL protein levels. BDNF also binds to nerve growth factor receptor (NGFR), also known as p75 neurotrophin receptor (p75NTR), albeit with a low-affinity [68,69]. MCR5 was also able to increase the levels of NGFR. These changes, jointly with the increase of the synaptic marker PSD95 levels that was demonstrated in SAMP8 mice after I_2_-IR ligand treatment, revealed that MCR5 promoted synaptic function, which is reported impaired in brain disorders.

**Figure 8:**
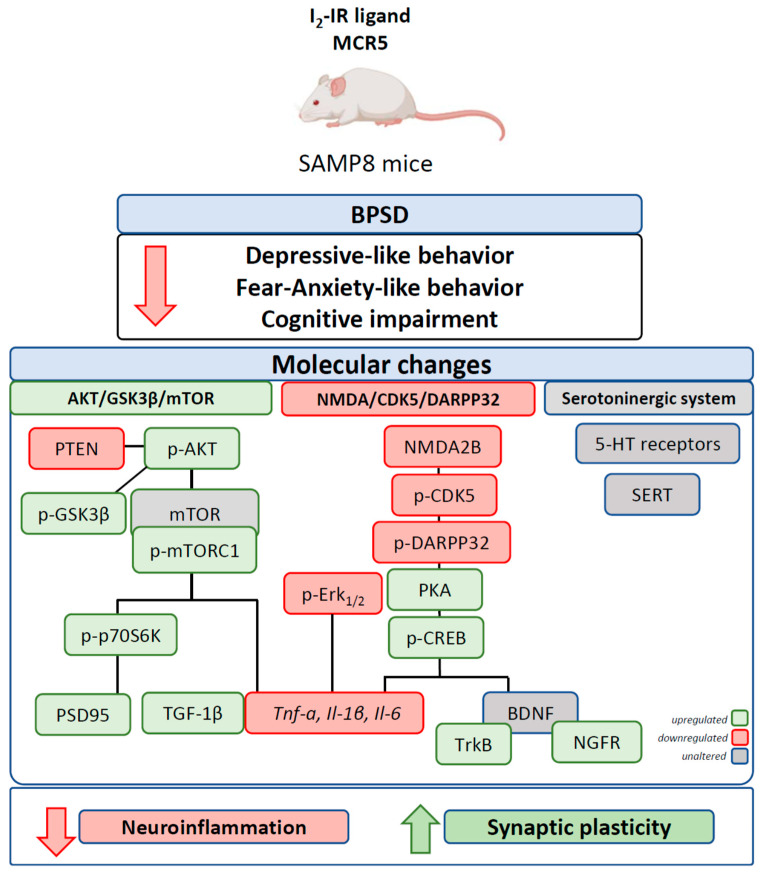
Cartoon illustrating a summary of behavioural, cognitive and molecular effects after MCR5 treatment in SAMP8 mice.

Altogether, these results demonstrated that MCR5 also plays a neuroprotective role against neurodegeneration induced through pathways associated with anxiety and depression, pointing out an alternative target for slowing down the disease progression (Figure 8).

## Supplementary Materials

The following are available online at https://www.mdpi.com/1999-4923/12/5/475/s1, Table S1: Antibodies used in Western blot studies, Table S2: Primers and probes used in qPCR studies, Table S3: Results of Novel object recognition test (NORT), and Object location test (OLT) Table S5: Parameters measured in the Open Field Test (OFT), Table S6: In Vitro Pharmacology: Binding Assays for MCR5 (10 μM) to 5-HT receptors and transporter.
