# Supplementary material for: Amelioration of BPSD-Like Phenotype and Cognitive Decline in SAMP8 Mice Model Accompanied by Molecular Changes after Treatment with I2-Imidazoline Receptor Ligand MCR5
Source: Pharmaceutics. 2020 May 23;12(5):475. doi: 10.3390/pharmaceutics12050475 (PMC7285228; doi:10.3390/pharmaceutics12050475)
Supplement: Supplementary file 1 [file pharmaceutics-12-00475-s001.pdf]

pharmaceutics

Supplementary Materials: Amelioration of BPSD-Like Phenotype and Cognitive Decline in SAMP8 Mice Model Accompanied by Molecular Changes after Treatment with I_2-Imidazoline Receptor Ligand MCR5

Foteini Vasilopoulou, Andrea Bagan, Sergio Rodriguez-Arevalo, Carmen Escolano, Christian Griñán-Ferré and Mercè Pallàs

Table S1. Antibodies used in Western blot studies.

Antibody	Host	Source/Catalog	WB Dilution
SERT	Goat	Abcam/ab130130	1:1000
p-AKT (Ser473)	Rabbit	Cell signaling/#D9E	1:1000
AKT	Rabbit	Cell signaling/#9272	1:1000
PTEN	Rabbit	Abcam/ab31392	1:1000
p-GSK3 (Ser9)	Rabbit	Cell signaling/#9336	1:1000
GSK3	Rabbit	Cell signaling/#9315	1:1000
p-mTOR	Rabbit	Santa Cruz/sc-293089	1:500
mTOR	Rabbit	Novus Biological/nb100-240	1:500
p-TORC1 (Ser 151)	Rabbit	Cell signaling/#3359	1:500
TORC1	Rabbit	Cell signaling/#2501	1:1000
p-p70S6K	Rabbit	Invitrogen/PA5-18093	1:1000
p70S6K	Goat	Invitrogen/44-920G	1:1000
TGF-1β	Rabbit	Cell Signaling/3711	1:1000
PKA a	Mouse	Santa Cruz/sc-28315	1:1000
p-NMDRA2B (Tyr1472)	Rabbit	Invitrogen/OPA1-04116	1:1000
NMDAR2B	Mouse	Santa Cruz/sc-365597	1:1000
p-DARPP32 (Thr75)	Rabbit	Cell Signaling/#2301	1:500
DARPP32	Mouse	Santa Cruz/sc-271111	1:1000
p-CDK5	Rabbit	Abcam/ab63550	1:1000
CDK5	Rabbit	Santa Cruz/sc-173	1:1000
p-CREB(Ser133)	Rabbit	Cell signaling/#9198	1:1000
ERK½	Rabbit	Cell Signaling/#9102	1:1000
p-ERK½ (Thr202/Tyr204)	Rabbit	Cell Signaling/#9101	1:1000
CREB	Rabbit	Cell signaling/9197	1:1000
TrkB	Rabbit	Santa Cruz/sc-8316	1:1000
NGFR	Mouse	Santa Cruz/sc-271708	
PSD95	Rabbit	Abcam/ab18258	1:1000
GAPDH	Mouse	Millipore/MAB374	1:5000
Actin	Mouse	Sigma-Aldrich/A5441	1:2500
Goat-anti-mouse HRP conjugated		Biorad/170-5047	1:5000
Goat-anti-rabbit HRP conjugated		Biorad/170-6515	1:5000
Donkey-anti-goat HRP conjugated		Santa Cruz/sc-2020	1:5000

Table S2. Primers and probes used in qPCR studies.

Target	Product Size (bp)	Forward Primer (5′-3′)	Reverse Primer (5′-3′)
5-HT1A	293	GGAGCGGGCACCAGCTTCGGAACA	CACTGTCTTCCTCTCACGGGCCAA
5-HT1B	125	AAGAAACTCATGGCCGCTAGGGAG	GCGTATCAGTTTGTGGAACGCTTG
5-HT2A	172	GGGTACCTCCCACCGACAT	AGGCCACCGGTACCCATAC
Tnf-α	157	TCGGGGTGATCGGTCCCCAA	TGGTTTGCTACGACGTGGGCT
Il-1β	179	ACAGAATATCAACCAACAAGTGATA TTCTC	GATTCTTTCCTTTGAGGCCCA
Il-6	189	ATCCAGTTGCCTTCTTGGGACTGA	TAAGCCTCCGACTTGTGAAGTGGT
β-actin	190	CAACGAGCGGTTCCGAT	GCCACAGGTTCCATACCCA

Table S3. Results of Novel object recognition test (NORT), and Object location test (OLT) in male mice at 10-month-old SR1, SP8 Ct mice groups and SP8 treated with I_2-IR ligand MCR5 (5mg/Kg) mice group. Summary of Discrimination Index (DI) from short-term memory (2h), summary of DI from long-term memory (24h), and summary of DI from OLT. *p < 0.05; **p < 0.01; ***p < 0.001; ****p < 0.0001 vs SR1-Ct.

**Summary NORT 2 h**		
**Group**	**Number of mice**	**DI (−1,1)**
SR1-Ct	11	0.258 ± 0.034**
SP8-Ct	11	0.010 ± 0.022
SP8-MCR5	14	0.274 ± 0.051***
**Summary NORT 24 h**		
**Group**	**Number of mice**	**DI (−1,1)**
SR1-Ct	11	0.260 ± 0.048***
SP8-Ct	11	0.032 ± 0.031
SP8-MCR5	14	0.299 ± 0.029***
**Summary OLT**		
**Group**	**Number of mice**	**DI (−1,1)**
SR1-Ct	11	0.267 ± 0.057***
SP8-Ct	11	-0.045 ± 0.035
SP8-MCR5	14	0.131 ± 0.039*

Table S4. Parameters measured in the Elevate plus maze (EPM). (n): number of events. Results are expressed as a mean ± Standard error of the mean (SEM). *p < 0.05; **p < 0.01; ***p < 0.001; ****p < 0.0001 vs SP8-MCR5 (5mg/Kg) $p < 0.05; $$p < 0.01; $$$$p < 0.0001 vs SR1-Ct.

	**SR1-Ct**	**SP8-Ct**	**SP8-MCR5 (5mg/Kg)**
Anxiety Index	1.11 ± 0.01**	1.08 ± 0.02**	0.95 ± 0.03
Locomotor Activity (cm)	977.42 ± 39.85	761.56 ± 42.70 $$ *	954.67 ± 45.07
Time spent in Open Arms (s)	25.31 ± 5.96****	43.52 ± 10.89***	99.02 ± 9.80
Time Spent in Closed Arms (s)	20.50 ± 2.49****	12.50 ± 1.21$ *** $	15.55 ± 0.92
Entries in Open Arms	13.90 ± 4.44*	10.75 ± 1.57*	17.64 ± 1.94
Rearings (n)	23.90 ± 1.25	12.25 ± 1.59** $$$$	19.60 ± 1.24
Groomings (n)	1.30 ± 0.29 $^{0.09}$	1.38 ± 0.18 $^{0.07}$	0.60 ± 0.27
Defecations (n)	0.70 ± 0.38	0.25 ± 0.16	0.20 ± 0.13
Urinations (n)	0.00 ± 0.00	0.0 ± 0.00	0.0 ± 0.00

Table S5. Parameters measured in the Open Field Test (OFT). (n): number of events. Results are expressed as a mean ± Standard error of the mean (SEM). *p < 0.05; **p < 0.01; ***p < 0.001; ****p < 0.0001 vs SP8-MCR5 (5mg/Kg). $p < 0.05 vs SR1-Ct.

	**SR1-Ct**	**SP8-Ct**	**SP8-MCR5 (5mg/Kg)**
Locomotor activity (cm)	1594.44±94.34**	1680.77 ± 104.53*	2036.58 ± 89.77
Mean Speed(cm/s)	5.32±0.31**	5.60±0.35*	6.62±0.26
Distance in Center (cm)	38.36±4.79	32.67±5.69*	62.95 ± 10.86
Entries in Center (n)	3.27±0.33***	4.30±0.76*	7.33±0.94
Time in Center (s)	4.83±0.69	3.52 ± 0.70	5.16 ± 0.77
Rearings (n)	20.73±2.03	14.45 ± 2.31**	26.42 ± 2.27
Grommings (n)	2.40 ± 0.31	1.18 ± 0.26 $	1.75 ± 0.25
Defecations (n)	1.55 ± 0.31	1.18 ± 0.44	0.75 ± 0.33
Urinations (n)	0.18 ± 0.18	0.0 ± 0.00	0.17 ± 0.11

Table S6. In Vitro Pharmacology: Binding Assays for MCR5 (10μM) to 5-HT receptors and transporter. Results showing an inhibition (or stimulation) lower than 50% are not considered significant and mostly attributable to variability of the signal around the control level.

**Receptor/Transporter**	**Source (Biological Susbtrate)**	**Ligand/ Ki (M)**	**% Inhibition of Control Specific Binding by MCR5 (10μM)**
5-HT1A	Human recombinant (HEK-293 cells)	[^{3}H]8-OHDPAT 3.9 × 10^{-10} M	0.2
5-HT1B	Human recombinant (Chem-1 (RBL) cells)	[^{3}H]GR125743 5.9 × 10^{-8} M	−7.0
5-HT2A	Human recombinant (HEK-293 cells)	[^{125}I](±)DOI 3.1 × 10^{-10} M	28.2
5-HT2B	Human recombinant (CHO cells)	[^{125}I](±)DOI 2.0 × 10^{-9} M	21.4
5-HT3	Human recombinant (CHO cells)	[^{3}H]BRL 43694 1.9 × 10^{-8} M	−10.8
5-HT transporter	Human recombinant (CHO cells)	[^{3}H]imipramine 1.2 × 10^{-9} M	−15.9
